# The buffering effect of perceived teacher support on students’ academic burnout in online learning: a mini review

**DOI:** 10.3389/fpsyg.2026.1919759

**Published:** 2026-09-04

**Authors:** Chen Li, Ze Wang, Nazgul Seifullinkyzy Kozhamkulova, Na Jiang, Xiaoyu Luo

**Affiliations:** 1 Al-Farabi Kazakh National University, Almaty, Kazakhstan; 2 Abai Kazakh National Pedagogical University, Almaty, Kazakhstan; 3 Shangrao Normal University, Shangrao, China

**Keywords:** academic burnout, buffering effect, higher education students, online learning, perceived teacher support

## Abstract

The widespread adoption of online learning in higher education has brought increased attention to academic burnout among university students. Although numerous studies have explored the manifestations, influencing factors and interventions of college students’ online academic burnout, few have consolidated and summarized the buffering effect of perceived teacher support. This Mini-Review, informed by the PRISMA 2020 framework, identified and analyzed 26 empirical studies retrieved from Web of Science and Scopus, extracting key data on research design, sample characteristics, and analytical approaches into a structured Excel matrix. This synthesis identifies three main findings. First, academic burnout in online settings is characterized by emotional exhaustion, heightened anxiety, loss of control, and a paradoxical imbalance between learning anxiety and enjoyment, shaped by both internal resources (psychological capital, self-efficacy, digital resilience) and external barriers (pedagogical, technological, and social constraints). Second, perceived teacher support is associated with reduced burnout through a direct protective pathway and three indirect mechanisms: enhancing academic self-efficacy, regulating emotional experiences (e.g., enjoyment, boredom, and anxiety), and facilitating acceptance of online learning (perceived usefulness and ease of use). Third, individual factors (e.g., psychological capital, digital resilience) and contextual conditions (e.g., peer support, technical environment) are tentatively proposed to moderate these associations, pending direct empirical testing. By integrating existing research and identifying gaps in the field, this review provides theoretical and practical insights for alleviating university students’ online academic burnout, optimizing teacher-student interaction in online settings, and building a supportive teaching system.

## 1 Introduction

The tension between inspiration and exhaustion in education, memorably captured in films like *Dead Poets Society* (Serey, 1992) and *The Paper Chase* (Trier, 2003), has taken on new dimensions in online learning. Screens blur life-study boundaries, inducing loss of control, isolation, and emotional exhaustion—academic burnout. This raises whether teacher support can still reach students through a screen.

Online learning has become mainstream in higher education, offering flexibility and personalized spaces (Dhawan, 2020; Greenhow et al., 2022; Hongsuchon et al., 2022). However, it also presents challenges, including reduced social interaction (Akpen et al., 2024), increased technical difficulties (Erlangga, 2022), reduced self-regulatory ability (Jin et al., 2023), and blurred boundaries between study and life (Conrad et al., 2022). These challenges may impose psychological and academic strain, contributing to academic burnout.

Teacher support deserves special attention among the above factors. Perceived teacher support refers to students’ belief that their teachers care about their learning and well-being, provide effective feedback, and offer help (An et al., 2022; Guo et al., 2023; Zhao and Yang, 2022). This form of support can also foster positive teacher-student relationships (Zhao and Yang, 2022). Compared with general social support, teacher support is more professional and directive, and may play a unique buffering role in alleviating academic burnout in online learning.

Academic burnout in online learning has attracted widespread attention from scholars (Basri et al., 2022; Huang et al., 2023; Liu and Cao, 2022; Wang et al., 2025; Yang G. et al., 2022). For example, Liu and Cao (2022) found that academic stress directly exacerbates burnout levels, with psychological resilience playing a partial mediating and buffering role. Basri et al. (2022) further indicated that academic burnout mediated the relationship between stress and learning outcomes, such that the positive effect of stress was weakened by burnout, while the buffering effect of resilience was limited, thereby leading to reduced learning outcomes.

Meanwhile, Wang et al. (2025) found that psychological resilience directly and positively predicted performance and satisfaction in online learning, and indirectly enhanced the learning experience by reducing academic burnout; conversely, insufficient psychological resilience exacerbated negative emotions and burnout. Moreover, from a longitudinal perspective, Huang et al. (2023) found that perceived social support indirectly alleviated academic burnout by promoting cognitive engagement, and that this effect strengthened over time, suggesting that teachers may play a similar role.

Several systematic reviews have synthesized evidence in related domains. For instance, Prananto et al. (2025) systematically reviewed the relationship between perceived teacher support and student engagement among higher education students, concluding that teacher support consistently predicts engagement across diverse contexts. However, their review did not examine academic burnout as an outcome variable, leaving the buffering role of teacher support against burnout unexplored. Similarly, Chong et al. (2025) synthesized factors contributing to student burnout across various student populations, identifying key individual and contextual antecedents; yet their review did not specifically address the protective function of teacher support in online learning environments.

Additionally, Tang et al. (2021) conducted a meta-analysis of intervention effects on learning burnout, confirming that targeted interventions can effectively reduce burnout, but their analysis did not isolate teacher support as a distinct protective factor, nor did it examine the mediating pathways through which such support operates. In sum, although existing reviews have illuminated important aspects of teacher support or academic burnout separately, few have consolidated the specific mechanisms through which perceived teacher support buffers academic burnout in the context of online higher education.

This gap is compelling given online learning’s unique challenges—reduced social presence, technical barriers, and blurred boundaries—which may intensify burnout and alter how teacher support is perceived. Without an integrated synthesis, researchers lack a coherent framework for leveraging teacher support to mitigate burnout online.

To address this gap, the present Mini-Review synthesizes evidence from 26 empirical studies to answer two research questions: (a) What are the current characteristics and influencing factors of academic burnout among university students in the online learning environment? (b) How is perceived teacher support associated with academic burnout among university students in such contexts, and what are its pathways?

By synthesizing fragmented evidence into a proposed conceptual model, this Mini-Review contributes a structured framework that clarifies the pathways through which teacher support may mitigate online academic burnout and offers actionable guidance for researchers, educators, and policymakers.

## 2 Methodology

The following subsections detail the literature search, screening, and data extraction procedures employed in this Mini-Review.

### 2.1 Search strategy and selection process

#### 2.1.1 Search strategy

The selection process was informed by the PRISMA 2020 statement (Page et al., 2021) for transparency. On May 15, 2026, we searched Scopus and Web of Science for peer-reviewed articles on perceived teacher support and online academic burnout, limited to title and abstract fields.

For perceived teacher support, we adopted the terms “perceived teacher support” and “teacher support” (Prananto et al., 2025). For academic burnout, we identified alternative keywords by consulting relevant published reviews (Chong et al., 2025; Tang et al., 2021). The burnout-related search terms were intentionally broad (e.g., “academic burnout,” “learning burnout,” “emotional exhaustion,” and related constructs) to maximize sensitivity and minimize the risk of missing relevant studies, as recommended for comprehensive searching in emerging research areas (Lefebvre et al., 2022). The full search strategy is detailed in Supplementary Table S1.

#### 2.1.2 Inclusion and exclusion criteria

We applied inclusion/exclusion criteria at three stages: database search, title/abstract screening, and full-text review.

The initial search yielded 150 records (61 from Web of Science, 89 from Scopus). After removing 49 duplicates, 101 records were retained for screening.

Two reviewers (LC and WZ) screened titles/abstracts, excluding 17 conference papers and 5 non-English articles; 79 remained. To avoid premature exclusion due to insufficient abstracts, no topic-based exclusions were applied.

- Pathway A (RQ1: burnout characteristics): Included empirical studies (quantitative, qualitative, or mixed) on university students’ academic burnout in online/blended contexts. Teacher support was not required.
- Pathway B (RQ2: buffering mechanisms): Included studies that quantitatively examined the teacher support–burnout relationship (or adjacent outcomes like engagement or satisfaction) in higher education samples, reporting verifiable estimates (e.g., correlations, regressions, path coefficients).

Reviews were excluded. Disagreements were resolved through discussion with a third reviewer (NSK).

**Figure 1:**
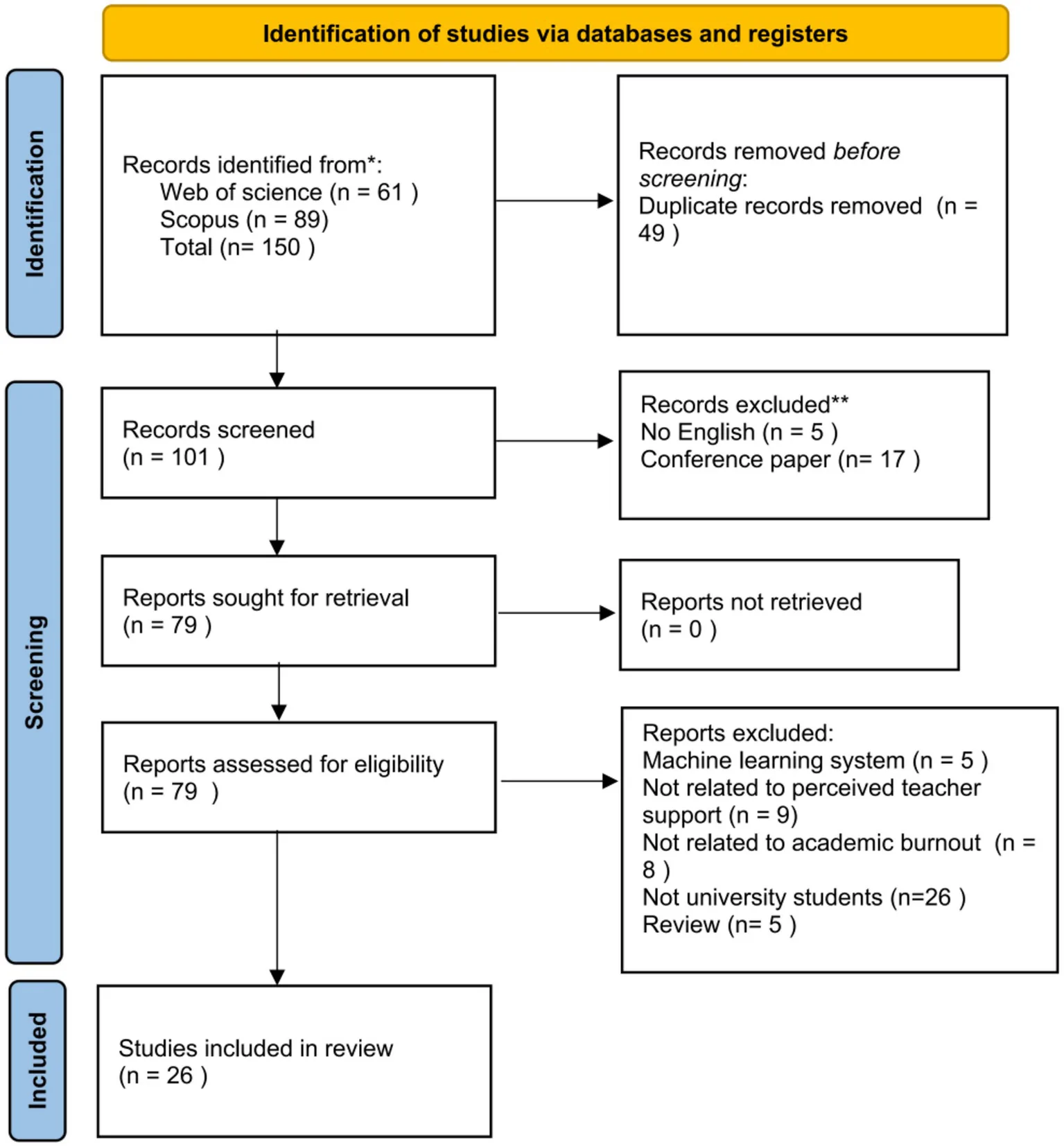
PRISMA 2020 flow diagram for the selection process of this mini-review. Format exclusions (non-English articles and conference papers) were applied during title/abstract screening; all substantive exclusions (machine learning systems, studies not addressing perceived teacher support or academic burnout, non-university-student samples, and review articles) were applied during full-text eligibility assessment, based on the dual-pathway criteria described in Section 2.1.2.

Applying these pathways, we excluded 22 articles not meeting either pathway’s criteria, 26 with non-higher-education samples, and 5 reviews, leaving 26 studies. Among these, 13 studies contributed to RQ1 and the remaining 13 to RQ2. Exclusion counts are detailed in the PRISMA flow diagram (Figure 1).

#### 2.1.3 Extraction and analysis

Two reviewers (LC and WZ) independently extracted data using a standardized Excel grid. The extracted fields included author(s), year, country, study design, sample characteristics, data collection methods, analytical techniques, and key findings. Discrepancies were resolved as above. The detailed descriptive information of the 26 included articles is presented in Supplementary Table S2.

As a Mini-Review, we did not conduct formal quality appraisal using standardized tools (e.g., MMAT), given our aim is to synthesize conceptual pathways rather than pool effect sizes. However, we interpret all findings with caution in the discussion.

**Figure 2:**
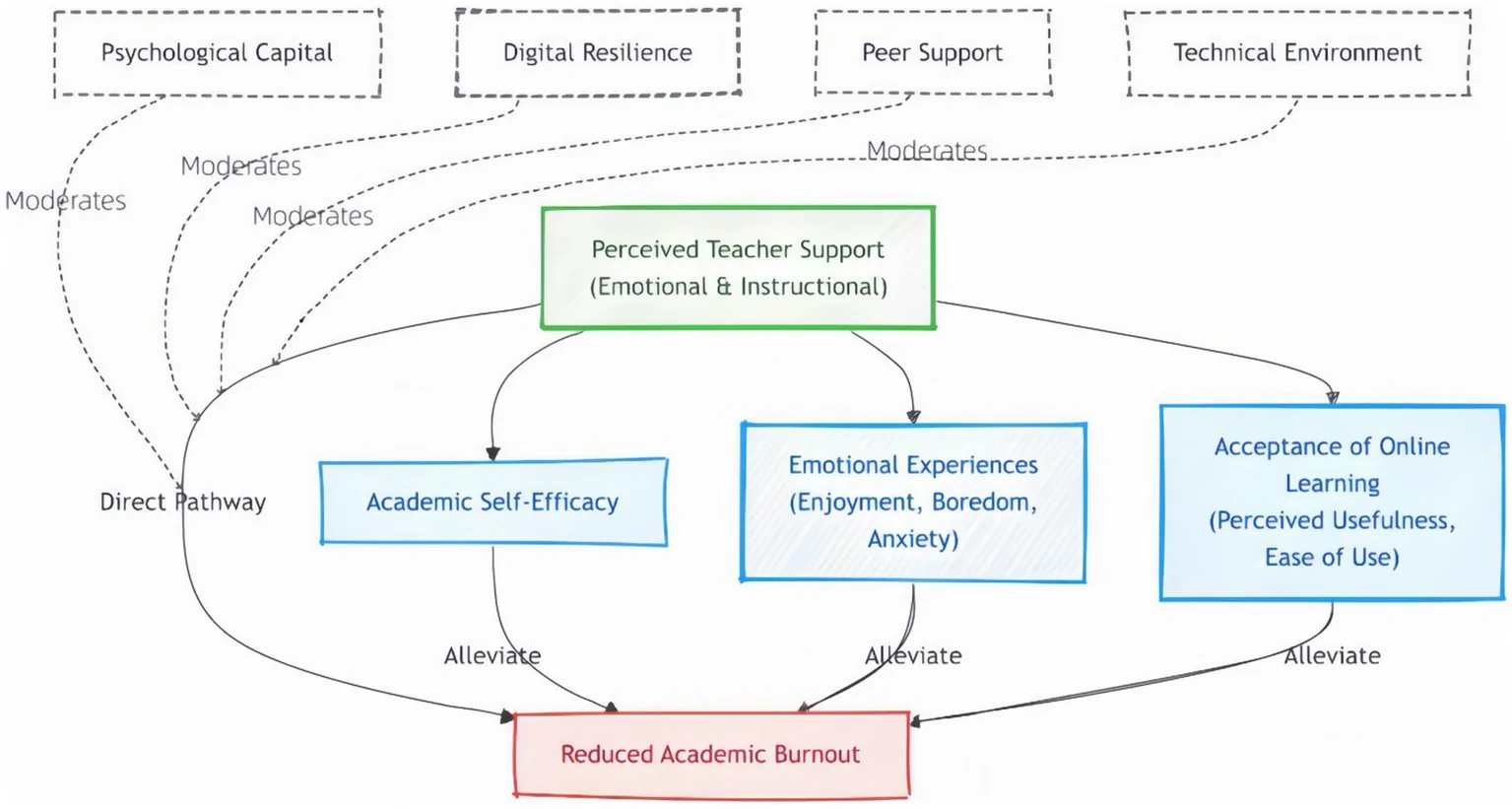
A proposed conceptual model of the pathways through which perceived teacher support is associated with students’ academic burnout in online learning. Solid lines represent hypothesized direct and indirect (mediating) pathways. Perceived teacher support is proposed to be directly associated with reduced academic burnout (Direct Pathway), and indirectly associated via three mediating mechanisms: enhancing academic self-efficacy, regulating emotional experiences (enjoyment, boredom, anxiety), and facilitating acceptance of online learning (perceived usefulness, ease of use). Dashed lines represent proposed moderating effects, with all moderator arrows terminating on the direct pathway between perceived teacher support and academic burnout, indicating that individual factors (psychological capital, digital resilience) and contextual factors (peer support, technical environment) are hypothesized to influence the strength of this association. The moderators are theoretically inferred from the broader literature rather than empirically tested within the synthesized corpus, and are depicted as dashed lines to indicate theoretical propositions. The model is synthesized from correlational and cross-sectional evidence and is intended as a conceptual framework for organizing existing findings and guiding future research, rather than as a confirmed causal model.

The extracted data were synthesized using a thematic approach. The same two reviewers independently reviewed the extracted findings to identify recurring patterns and relationships related to the research questions. Initial codes were grouped into broader themes through iterative discussion, resulting in the organizational structure of Section 3 (characteristics and influencing factors of burnout; buffering pathways of teacher support), and the conceptual model (Figure 2) was subsequently developed to integrate these themes into a coherent framework. All thematic decisions were reviewed and confirmed by a third reviewer (NSK) to ensure consistency.

## 3 Results

This section synthesizes the findings of the 26 included studies to answer the two research questions guiding this Mini-Review. The findings are organized around (a) the characteristics and influencing factors of academic burnout in online learning, and (b) the buffering pathways of perceived teacher support as synthesized in a conceptual model (see Figure 2).

### 3.1 Characteristics and influencing factors of academic burnout

Academic burnout in online settings manifests primarily as emotional exhaustion, loss of control, and persistent anxiety accompanied by psychological distress (Kee, 2021; Patil et al., 2022). Kee (2021) found that pandemic-induced uncertainty blurred academic-daily life boundaries, depriving students of independent study space and intensifying their sense of losing control. Patil et al. (2022) further reported that prolonged isolation led nearly 60% of students to lack confidence in their future clinical skills.

Notably, Ramírez-Martinez et al. (2024) observed that anxiety levels peaked not during lockdown but in the first post-return face-to-face semester, coinciding with a progressive decline in perceived emotional support. Schwartz et al. (2025) documented that 89.5% of students experienced strong social isolation, with screen fatigue and weak community belonging directly reducing engagement. Extending these findings on emotional distress, Nusrat (2022) reported that 55.2% of university students engaged in online learning experienced severe psychological distress, and identified that coping strategies such as active coping, seeking emotional support, and self-blame significantly influenced the management of such distress.

Beyond emotional exhaustion, Kamaludin and Sundarasen (2023) synthesized pedagogical, technical, and social barriers that collectively generated physical and mental stress. Maican and Cocorada (2021) identified an imbalance between learning anxiety and enjoyment—lower-achieving students paradoxically reported higher enjoyment of low-difficulty materials, which potentially signaled early burnout. Huang et al. (2026) noted that technical difficulties and insufficient peer interaction exacerbated foreign language communication anxiety in MOOCs.

The formation of academic burnout results from the interplay of internal and external factors. Internally, psychological capital negatively predicted learning burnout, with learning engagement playing a partial mediating role (Fu and Qiu, 2024). Li et al. (2025) identified five core themes of digital resilience—understanding digital threats, knowing coping strategies, learning skills, overcoming digital stress, and adapting to the environment.

Externally, Kamaludin and Sundarasen (2023) confirmed that pedagogical challenges (continuous assessments and excessive workloads), technical barriers (unreliable internet and device shortages), and social difficulties (insufficient family support and unconducive home environments) generated physical and psychological stress. Adarkwah and Huang (2023) found that while blended learning alleviated purely online difficulties, academic anxiety persisted under multi-track systems. Similarly, Smith and Werse (2025) found via autoethnography that flexible scheduling, emotional support, and close communication reduced dissertation-related stress in an online doctoral program, highlighting external support’s role in mitigating academic strain. Yang D. L. et al. (2022) classified the required forms of support into autonomy, competence, and relatedness, and identified their absence as a key external trigger for burnout.

In summary, academic burnout is characterized by emotional exhaustion, escalating anxiety, psychological distress, and an anxiety-enjoyment imbalance, shaped by internal resources (psychological capital, self-efficacy, digital resilience) and external conditions (pedagogical, technical, and social support).

### 3.2 Buffering pathways of perceived teacher support

Based on the 26 included studies, Figure 2 presents a conceptual model that integrates direct pathways, mediating mechanisms, and moderating conditions. The model delineates the proposed direct association between perceived teacher support and academic burnout, as well as three indirect pathways—academic self-efficacy, emotional experiences, and acceptance of online learning—and acknowledges that individual and contextual factors may moderate these pathways.

#### 3.2.1 Direct effects

Perceived teacher support is associated with reduced online learning burnout, though not all studies modeled it directly. Sun L. N. et al. (2025) found that teacher emotional support significantly and negatively predicted academic burnout. Abdullah et al. (2022) reported that teacher support predicted academic performance more strongly than students’ well-being, but did not estimate a support-to-burnout path. Tauchid (2025) showed that combined teacher and peer support was associated with reduced online speaking anxiety and improved proficiency, without directly modeling burnout. Among these, only Sun L. N. et al. (2025) directly estimated the perceived teacher support–academic burnout relationship.

#### 3.2.2 Indirect (mediating) mechanisms

Several cited studies examined adjacent outcomes (e.g., engagement, satisfaction) rather than burnout directly; the actual outcome is noted below, and these are interpreted as indicative rather than confirmatory.

First, academic self-efficacy serves as a mediator. He et al. (2024) found that perceived teacher emotional support indirectly enhanced online academic engagement (not burnout) by strengthening academic self-efficacy, accounting for 28.85% of the total effect. Yang G. et al. (2022) showed that teacher emotional support reduced online burnout via self-efficacy, which also mediated burnout and learning satisfaction, supporting the self-efficacy pathway in the support–burnout link. Figueredo et al. (2023) further revealed that burnout and engagement jointly mediated the relationship between teacher support and academic satisfaction.

Second, emotional experiences constitute another pathway, encompassing positive (e.g., enjoyment) and negative (e.g., boredom, anxiety) emotions. Li et al. (2024) found that teacher support partially offset foreign language anxiety’s negative effect on engagement. Additionally, Liu and Zhou (2024) clarified that teacher emotional support operated sequentially through enjoyment and boredom—with enjoyment alone exhibiting a stronger mediating effect than the serial pathway—rather than directly predicting engagement.

Third, acceptance of online learning functions as an additional mediator. He et al. (2023) found that perceived educational support positively influenced both perceived usefulness and ease of use, while perceived emotional support uniquely enhanced ease of use, jointly promoting continuance intention. Robinson (2025) confirmed that instructional support, alongside learning readiness and peer support, fostered online learning acceptance, which was inversely associated with burnout (the only study in this pathway using burnout as the direct outcome).

Moderating conditions (theoretically inferred). Based on the broader literature, individual factors (psychological capital, digital resilience) and contextual factors (peer learning atmosphere, technical-environmental support) may moderate the teacher support-burnout association, though these interactions were not formally tested in the included studies (Fu and Qiu, 2024; Li et al., 2025; Kamaludin and Sundarasen, 2023). None of the 26 studies formally tested these effects. These moderators are depicted as dashed lines in Figure 2 to indicate theoretical propositions, pending direct empirical testing (see Section 4.4).

#### 3.2.3 Intervention evidence

Targeted pedagogical interventions incorporating teacher support have been examined. Active learning and programming activities were associated with boosted motivation, self-efficacy, and performance, and reduced anxiety (Ariza, 2023). Performance-based assessment was associated with improved academic resilience and motivation—with stronger effects virtually than face-to-face—and teacher support amplified this benefit (Yaghoubi et al., 2025). Additionally, structured course adjustments (e.g., controlled reading time, limited test attempts) were associated with enhanced intrinsic motivation and online engagement (Sun J. C. Y. et al., 2025).

## 4 Discussion

### 4.1 Summary of main findings

This Mini-Review synthesized 26 studies on perceived teacher support and online academic burnout. Burnout is characterized by emotional exhaustion, heightened anxiety, loss of control, and an anxiety-enjoyment imbalance, shaped by internal resources (psychological capital, self-efficacy, digital resilience) and external barriers (pedagogical, technological, and social constraints). Perceived teacher support is associated with reduced burnout through direct and three indirect pathways—enhancing self-efficacy, regulating emotional experiences (enjoyment, boredom, anxiety), and facilitating acceptance of online learning. Individual and contextual factors are theoretically proposed to moderate these associations, though these interactions were not directly tested. Figure 2 presents this conceptual model.

### 4.2 Critical appraisal of the evidence

Several methodological considerations warrant caution. Most included studies employed cross-sectional designs with self-reported measures, precluding causal inference; reverse causality remains plausible. Measurement heterogeneity among studies complicates direct comparisons. Most samples were drawn from specific national contexts (e.g., China, Malaysia, Indonesia), limiting generalizability. As a Mini-Review, we did not conduct formal quality appraisal, meaning studies with varying rigor were given equal descriptive weight.

Additionally, much of the mediation evidence in Section 3.2 uses engagement, satisfaction, or continuance intention rather than burnout as the outcome. Though theoretically linked to burnout within the Job Demands-Resources and Self-Determination Theory frameworks, these are non-equivalent constructs; readers should interpret such findings as indicative rather than confirmatory. Future studies should model teacher support effects on burnout as the focal outcome.

### 4.3 Theoretical and practical implications

Theoretically, this review contributes a conceptual model integrating direct, mediating, and moderating pathways. Unlike prior reviews examining teacher support-engagement (Prananto et al., 2025) or burnout factors (Chong et al., 2025) separately, this synthesis specifies how teacher support buffers burnout online. By delineating three mediating pathways, the model extends self-determination theory and the job demands-resources model to online higher education, positioning teacher support as an external resource that enhances self-efficacy and reshapes affective responses. Including anxiety alongside enjoyment and boredom addresses a conceptual gap, underscoring its central role.

Practically, teacher emotional support—empathy and responsiveness—emerges as a consistent protective factor; institutions should train instructors accordingly. Teacher support should enhance self-efficacy, regulate emotions, and facilitate online learning acceptance. Given the moderating roles of peers and technical environment, a holistic teaching-peer-infrastructure approach is likely most effective.

### 4.4 Limitations and future directions

We acknowledge that restricting the search to Web of Science and Scopus may have excluded relevant studies from other databases (e.g., CNKI, ERIC, ProQuest), potentially introducing “false negatives” (Hiver et al., 2024). Second, excluding non-English and non-peer-reviewed literature may have excluded valuable findings. Third, as a Mini-Review, we did not conduct formal quality appraisal. Fourth, the cross-sectional evidence also constrains causal interpretations.

Future research should include additional databases, use longitudinal and experimental designs, expand geographic diversity, explore synergies among support types, and incorporate qualitative methods. Critically, the moderating effects proposed in Figure 2 require direct empirical testing (e.g., multiple regression with interaction terms or multi-group SEM), as these interactions were not formally tested in the synthesized corpus.

## 5 Conclusion

This Mini-Review synthesized 26 studies on perceived teacher support and online academic burnout. Burnout manifests as emotional exhaustion, heightened anxiety, loss of control, and an anxiety-enjoyment imbalance, shaped by internal resources and external barriers. Perceived teacher support is associated with reduced burnout directly and via three indirect mechanisms—enhancing self-efficacy, regulating emotions, and facilitating online learning acceptance—with individual and contextual factors proposed as moderators, though untested. The proposed conceptual model integrates these pathways into a coherent framework that synthesizes fragmented literature and provides theoretical direction and practical guidance for designing supportive online learning environments.

Given the predominance of cross-sectional evidence, caution is warranted in causal interpretations. Future longitudinal, experimental, and cross-cultural research is needed to test the proposed model. By systematically synthesizing available evidence, this Mini-Review offers a structured foundation for researchers, educators, and policymakers addressing academic burnout in online higher education.

## References

[ref1] Abdullah N. A. Abu Shamsi N. Jenatabadi H. S. Ng B. K. Mentri K. A. C. (2022). Factors affecting undergraduates' academic performance during COVID-19: fear, stress and teacher-parents' support. Sustainability 14, 1–12. doi: 10.3390/su14137694

[ref2] Adarkwah M. A. Huang R. H. (2023). Blended learning for the "multi-track" undergraduate students in Ghana in an adverse era. Sci. Afr. 21:e01772. doi: 10.1016/j.sciaf.2023.e01772, 37363048 PMC10281694

[ref3] Akpen C. N. Asaolu S. Atobatele S. Okagbue H. Sampson S. (2024). Impact of online learning on student's performance and engagement: a systematic review. Discov. Educ. 3, 1–15. doi: 10.1007/s44217-024-00253-0

[ref4] An F. Yu J. Xi L. (2022). Relationship between perceived teacher support and learning engagement among adolescents: mediation role of technology acceptance and learning motivation. Front. Psychol. 13:992464. doi: 10.3389/fpsyg.2022.992464, 36248506 PMC9562933

[ref5] Ariza J. A. (2023). Bringing active learning, experimentation, and student-created videos in engineering: a study about teaching electronics and physical computing integrating online and mobile learning. Comput. Appl. Eng. Educ. 31, 1723–1749. doi: 10.1002/cae.22673

[ref6] Basri S. Hawaldar I. T. Nayak R. Rahiman H. U. (2022). Do academic stress, burnout and problematic internet use affect perceived learning? Evidence from India during the COVID-19 pandemic. Sustainability 14, 1–18. doi: 10.3390/su14031409

[ref7] Chong L. Z. Foo L. K. Chua S.-L. (2025). Student burnout: a review on factors contributing to burnout across different student populations. Behav. Sci. 15, 1–12. doi: 10.3390/bs15020170, 40001801 PMC11852093

[ref8] Conrad C. Deng Q. Caron I. Shkurska O. Skerrett P. Sundararajan B. (2022). How student perceptions about online learning difficulty influenced their satisfaction during Canada's Covid-19 response. Br. J. Educ. Technol. 53, 534–557. doi: 10.1111/bjet.13206, 35600419 PMC9111658

[ref9] Dhawan S. (2020). Online learning: a panacea in the time of COVID-19 crisis. J. Educ. Technol. Syst. 49, 5–22. doi: 10.1177/0047239520934018

[ref10] Erlangga D. T. (2022). Student problems in online learning: solutions to keep education going on. J. Engl. Lang. Teach. Learn. 3, 21–26.

[ref11] Figueredo J. M. Molero-Jurado M. D. Vico-Sánchez M. F. Alonso-Delgado S. H. Rosales-Jiménez J. J. Torres-Rojas M. C. . (2023). Antecedents and mediators of academic satisfaction in virtual vocational training. Eur. J. Investig. Health Psychol. Educ. 13, 2498–2515. doi: 10.3390/ejihpe13110174, 37998064 PMC10670857

[ref12] Fu L. Qiu Y. (2024). Contributions of psychological capital to the learning engagement of Chinese undergraduates in blended learning during the prolonged COVID-19 pandemic: the mediating role of learning burnout and the moderating role of academic buoyancy. Eur. J. Psychol. Educ. 39, 837–860. doi: 10.1007/s10212-023-00759-5

[ref13] Greenhow C. Graham C. R. Koehler M. (2022). Foundations of online learning: challenges and opportunities. Educ. Psychol. 57, 131–147. doi: 10.1080/00461520.2022.2090364

[ref14] Guo Q. Samsudin S. Yang X. Gao J. Ramlan M. A. Abdullah B. . (2023). Relationship between perceived teacher support and student engagement in physical education: a systematic review. Sustainability 15, 1–18. doi: 10.3390/su15076039

[ref15] He L. Feng L. Ding J. (2024). The relationship between perceived teacher emotional support, online academic burnout, academic self-efficacy, and online English academic engagement of Chinese EFL learners. Sustainability 16, 1–20. doi: 10.3390/su16135542

[ref16] He S. Jiang S. W. Zhu R. L. Hu X. (2023). The influence of educational and emotional support on e-learning acceptance: an integration of social support theory and TAM. Educ. Inf. Technol. 28, 11145–11165. doi: 10.1007/s10639-023-11648-1, 36818430 PMC9926416

[ref17] Hiver P. Al-Hoorie A. H. Vitta J. P. Wu J. (2024). Engagement in language learning: a systematic review of 20 years of research methods and definitions. Lang. Teach. Res. 28, 201–230. doi: 10.1177/13621688211001289

[ref18] Hongsuchon T. Emary I. M. E. Hariguna T. Qhal E. M. A. (2022). Assessing the impact of online-learning effectiveness and benefits in knowledge management, the antecedent of online-learning strategies and motivations: an empirical study. Sustainability 14, 1–16. doi: 10.3390/su14052570

[ref19] Huang C. Tu Y. He T. Han Z. Wu X. (2023). Longitudinal exploration of online learning burnout: the role of social support and cognitive engagement. Eur. J. Psychol. Educ. 39, 361–388. doi: 10.1007/s10212-023-00693-6, 40479015 PMC10025060

[ref20] Huang W. Q. Yang P. Z. Shen H. Z. Yang H. Z. (2026). How do they stack up? Perceived communicative competence, foreign language anxiety, teacher support, and classroom environment in EFL learners' willingness to communicate in MOOCs. ReCALL 38, 1–17. doi: 10.1017/S0958344026100470, 41292463

[ref21] Jin S.-H. Im K. Yoo M. Roll I. Seo K. (2023). Supporting students’ self-regulated learning in online learning using artificial intelligence applications. Int. J. Educ. Technol. High. Educ. 20, 1–21. doi: 10.1186/s41239-023-00406-5, 38164791

[ref22] Kamaludin K. Sundarasen S. (2023). COVID-19 and online distance learning in Malaysia: a blessing or a curse? Front. Educ. 8, 1–12. doi: 10.3389/feduc.2023.1062219

[ref23] Kee C. E. (2021). The impact of COVID-19: graduate students’ emotional and psychological experiences. J. Hum. Behav. Soc. Environ. 31, 476–488. doi: 10.1080/10911359.2020.1855285

[ref24] Lefebvre C. Glanville J. Briscoe S. Littlewood A. Marshall C. Metzendorf M. I. . (2022). “Searching for and selecting studies,” in Cochrane Handbook for Systematic Reviews of Interventions, eds. Higgins J. P. T. Thomas J. Chandler J. Cumpston M. Li T. Page M. J. . (Chichester, West Sussex, England: Cochrane).

[ref25] Li F. F. Ma Q. P. Yang C. X. Zhong M. Y. (2025). Investigating key elements of digital resilience among nursing undergraduates: a qualitative study. Front. Med. 11:1452580. doi: 10.3389/fmed.2024.1452580, 39959609 PMC11825263

[ref26] Li X. M. Zhang F. L. Duan P. Yu Z. G. (2024). Teacher support, academic engagement and learning anxiety in online foreign language learning. Br. J. Educ. Technol. 55, 2151–2172. doi: 10.1111/bjet.13430

[ref27] Liu Y. Cao Z. (2022). The impact of social support and stress on academic burnout among medical students in online learning: the mediating role of resilience. Front. Public Health 10:938132. doi: 10.3389/fpubh.2022.938132, 35937240 PMC9355500

[ref28] Liu Q. Zhou W. (2024). The impact of teachers' emotional support on EFL learners' online learning engagement: the role of enjoyment and boredom. Acta Psychol. 250:104504. doi: 10.1016/j.actpsy.2024.104504, 39342900

[ref29] Maican M. A. Cocorada E. (2021). Online foreign language learning in higher education and its correlates during the COVID-19 pandemic. Sustainability 13, 1–21. doi: 10.3390/su13020781

[ref30] Nusrat E. M. (2022). Association between psychological distress and coping strategies among students engaged in online learning. PLoS One 17, 1–22. doi: 10.1371/journal.pone.0270877 PMC9249243 35776698

[ref31] Page M. J. McKenzie J. E. Bossuyt P. M. Boutron I. Hoffmann T. C. Mulrow C. D. . (2021). The PRISMA 2020 statement: an updated guideline for reporting systematic reviews. BMJ 372, 1–9. doi: 10.1136/bmj.n71, 33782057 PMC8005924

[ref32] Patil S. S. Hasamnis A. A. Chinna K. Wei N. G. W. Min B. N. R. (2022). Perceived impact of the prolonged COVID-19 pandemic on Malaysian medical students: a descriptive, cross-sectional study. Med. J. Dr. D.Y. Patil Vidyapeeth 15, S60–S64. doi: 10.4103/mjdrdypu.mjdrdypu_180_22

[ref33] Prananto K. Cahyadi S. Lubis F. Y. Hinduan Z. R. (2025). Perceived teacher support and student engagement among higher education students–a systematic literature review. BMC Psychol. 13, 1–22. doi: 10.1186/s40359-025-02412-w, 39934874 PMC11817619

[ref34] Ramírez-Martinez F. R. Villanos M. T. Sharma S. Leiner M. (2024). Variations in anxiety and emotional support among first-year college students across different learning modes (distance and face-to-face) during COVID-19. PLoS One 19, 1–14. doi: 10.1371/journal.pone.0285650, 38451887 PMC10919625

[ref35] Robinson L. Jr. (2025). Exploring determinants of online learning acceptance: the role of readiness, peer support, and instructional support. Electron. J. E-Learn. 23, 130–142. doi: 10.34190/ejel.23.2.4048

[ref36] Schwartz W. A. H. Merchant T. S. Sturla I. Neubauer L. C. (2025). Digital inequities and emotional resilience: understanding the impact of the COVID-19 pandemic on preclinical medical students. Front. Med. 12:1559536. doi: 10.3389/fmed.2025.1559536, 40575572 PMC12198243

[ref37] Serey T. T. (1992). Carpe diem: lessons about life and management from dead poets society. J. Manag. Educ. 16, 374–381. doi: 10.1177/105256299201600309

[ref38] Smith J. Werse N. R. (2025). Time and doctoral degrees wait for no one: an autoethnography on the creation of a culture of support in an online EdD program amidst a global pandemic. Inf. Learn. Sci. 126, 245–258. doi: 10.1108/ILS-10-2023-0152

[ref39] Sun J. C. Y. Lin C. T. Chang W. L. (2025). Are online behavioural characteristics effective predictors of intrinsic motivation and user engagement in the online learning environment? Australas. J. Educ. Technol. 41, 36–51. doi: 10.14742/ajet.10526

[ref40] Sun L. N. Ma R. S. Du C. H. Zhao J. M. Yang Y. L. Zhang X. G. (2025). A study on the impact of perceived teacher emotional support on university students' online learning engagement: the mediating role of academic burnout. Front. Psychol. 16:1625857. doi: 10.3389/fpsyg.2025.1625857, 40837254 PMC12361205

[ref41] Tang L. Zhang F. Yin R. Fan Z. (2021). Effect of interventions on learning burnout: a systematic review and meta-analysis. Front. Psychol. 12, 1–13. doi: 10.3389/fpsyg.2021.645662, 33716914 PMC7952745

[ref42] Tauchid A. (2025). Teacher and peer support as key factors in EFL learners' speaking anxiety and proficiency in online learning environments. Balt. J. Engl. Lang. Lit. Cult. 15, 125–142. doi: 10.22364/BJELLC.15.2025.09

[ref43] Trier J. (2003). Inquiring into ‘techniques of power’with preservice teachers through the ‘school film’the paper chase. Teach. Teach. Educ. 19, 543–557. doi: 10.1016/S0742-051X(03)00051-9

[ref44] Wang S. Wang Y. Zhao L. (2025). Effects of psychological resilience on online learning performance and satisfaction among undergraduates: the mediating role of academic burnout. Asia-Pac. Educ. Res. 34, 395–409. doi: 10.1007/s40299-024-00862-1

[ref45] Yaghoubi M. Gilakjani A. P. Abbasian G. R. (2025). The effect of performance-based assessment on academic resilience, motivation, and teacher support in virtual versus non-virtual classes. Lang. Test. Asia 15, 1–32. doi: 10.1186/s40468-025-00385-6

[ref46] Yang G. Sun W. Jiang R. (2022). Interrelationship amongst university student perceived learning burnout, academic self-efficacy, and teacher emotional support in China’s English online learning context. Front. Psychol. 13:829193. doi: 10.3389/fpsyg.2022.829193, 35360629 PMC8963801

[ref47] Yang D. L. Tang Y. M. Hayashi R. Ra S. Lim C. P. (2022). Supporting inclusive online higher education in developing countries: lessons learnt from Sri Lanka's university closure. Educ. Sci. 12, 1–11. doi: 10.3390/educsci12070494

[ref48] Zhao Y. Yang L. (2022). Examining the relationship between perceived teacher support and students’ academic engagement in foreign language learning: enjoyment and boredom as mediators. Front. Psychol. 13:987554. doi: 10.3389/fpsyg.2022.987554, 36204761 PMC9530908

