# Supplementary material for: The buffering effect of perceived teacher support on students’ academic burnout in online learning: a mini review
Source: Front Psychol. 2026 Sep 4;17:1919759. doi: 10.3389/fpsyg.2026.1919759 (PMC13588674; doi:10.3389/fpsyg.2026.1919759)
Supplement: Supplementary file 1 [file Table_1.DOCX]

Search Strategy

**Table 1**

*Search terms used in the Web of Science and Scopus for studies related to perceived teacher support and academic burnout in online learning among university students.*

| Step | Terms | Results |
| --- | --- | --- |
| Database: Web of science | | |
| 1 | TS= (“online learning” OR “e-learning” OR “remote learning” OR “distance learning”) | 86,155 |
| 2 | TS= (“**academic burnout” OR “academic burden” OR “student burnout” OR “learning burnout” OR “study burnout” OR “burnout” OR “fatigue” OR “exhaustion” OR “weariness” OR “academic burden” OR “scholastic stress” OR “cognitive exhaustion”** OR “stress” OR “anxiety” OR “academic stress” OR “student academic stress” OR “student anxiety” OR “academic anxiety” OR “learning stress” OR “study pressure”) | 3,591,560 |
| 3 | TS= (“perceived teacher support” OR “teacher support” OR “teacher-student relationship” OR “teacher autonomy support” OR “instructional support” OR “emotional support” OR “affective support” OR “autonomy support” OR “relatedness support” OR “competence support” OR “supportive teaching”) | 25,955 |
| 4 | TS= (“university students” OR “college students” OR “undergraduates” OR “postgraduates” OR “learner” OR “Student” OR “students” OR “undergraduate students” OR “undergraduate student” OR “undergraduate” OR “university student” OR “college student” OR “postgraduate students” OR “postgraduate student” OR “higher education students” OR “higher education student”) | 1,373,293 |
| 5 | 1 AND 2 AND 3 AND 4 | 61 |
| Database: Scopus | | |
| 1 | TITLE-ABS-KEY (“online learning” OR “e-learning” OR “remote learning” OR “distance learning”) | 216,409 |
| 2 | TITLE-ABS-KEY (“**academic burnout” OR “academic burden” OR “student burnout” OR “learning burnout” OR “study burnout” OR “burnout” OR “fatigue” OR “exhaustion” OR “weariness” OR “academic burden” OR “scholastic stress” OR “cognitive exhaustion”** OR “stress” OR “anxiety” OR “academic stress” OR “student academic stress” OR “student anxiety” OR “academic anxiety” OR “learning stress” OR “study pressure”) | 4,896,944 |
| 3 | TITLE-ABS-KEY (“perceived teacher support” OR “teacher support” OR “teacher-student relationship” OR “teacher autonomy support” OR “instructional support” OR “emotional support” OR “affective support” OR “autonomy support” OR “relatedness support” OR “competence support” OR “supportive teaching”) | 34,811 |
| 4 | TITLE-ABS-KEY (“university students” OR “college students” OR “undergraduates” OR “postgraduates” OR “learner” OR “Student” OR “students” OR “undergraduate students” OR “undergraduate student” OR “undergraduate” OR “university student” OR “college student” OR “postgraduate students” OR “postgraduate student” OR “higher education students” OR “higher education student”) | 2,248,362 |
| 5 | 1 AND 2 AND 3 AND 4 | 89 |
